# Supplementary material for: Cross-neutralizing and potent human monoclonal antibodies against historical and emerging H5Nx influenza viruses
Source: Nat Microbiol. 2025 Oct 14;10(11):2903–18. doi: 10.1038/s41564-025-02137-x (PMC12578633; doi:10.1038/s41564-025-02137-x)
Supplement: Supplementary file 1 — Supplementary Figs. 1–5, and Tables 1 and 3–5. [file 41564_2025_2137_MOESM1_ESM.pdf]

nature microbiology

Supplementary information

https://doi.org/10.1038/s41564-025-02137-x

Cross-neutralizing and potent human monoclonal antibodies against historical and emerging H5Nx influenza viruses

In the format provided by the authors and unedited

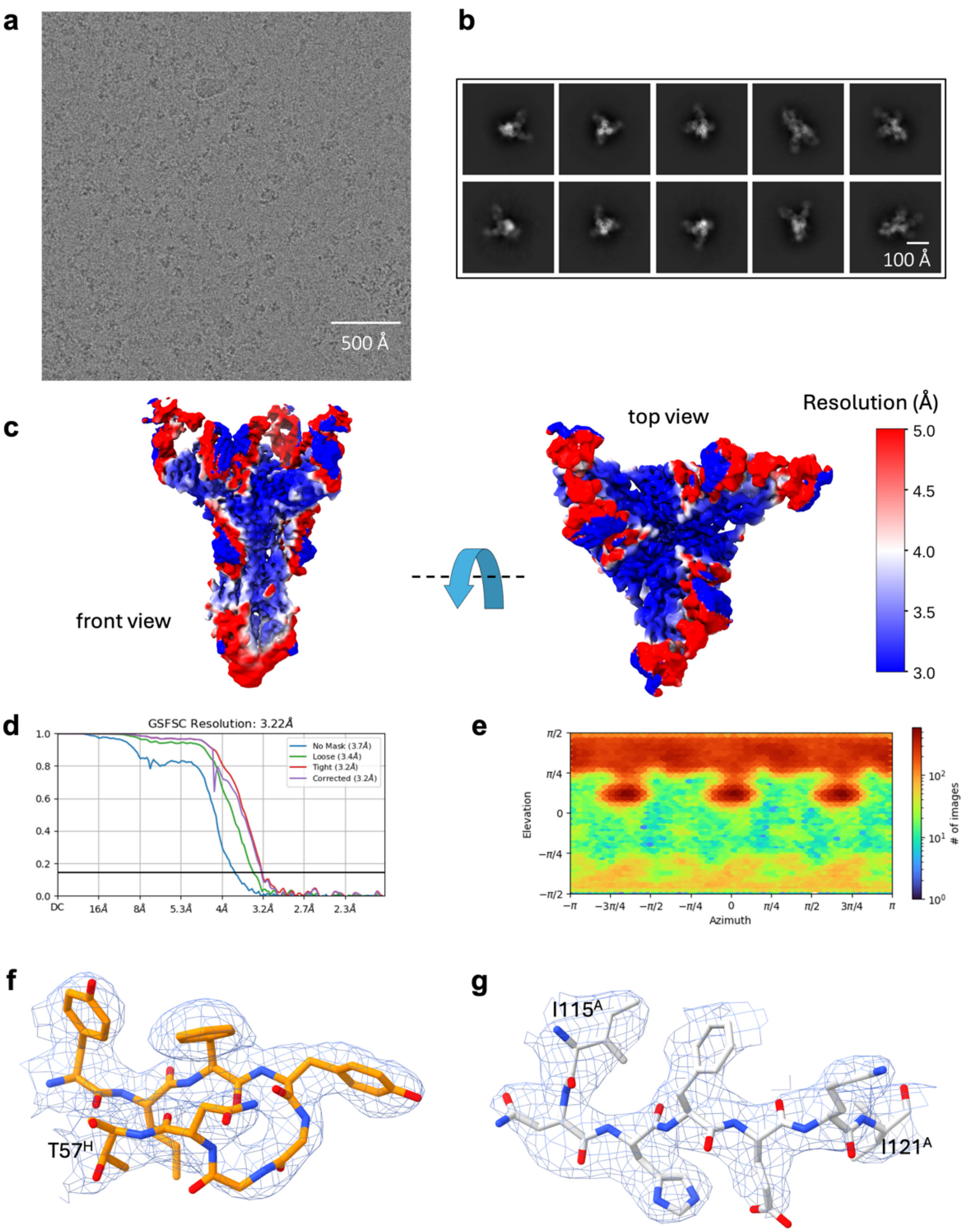

Supplementary Figure 1. Cryo-EM validation of HA in complex with 310-12D03.

a, Representative micrograph. **b**, Representative 2D class averages. **c**, The local resolution estimation of the reconstructed map. **d**, GSFSC resolution curves and **e**, heatmap showing the orientation of the particles used for reconstruction and refinement. **f**, Exemplary cryo-EM map density is shown for select residues of the Fab heavy chain involved in complex formation. **g**, Exemplary cryo-EM density of the residues in HA, chain A. The contour level is 12σ in both panels f and g.

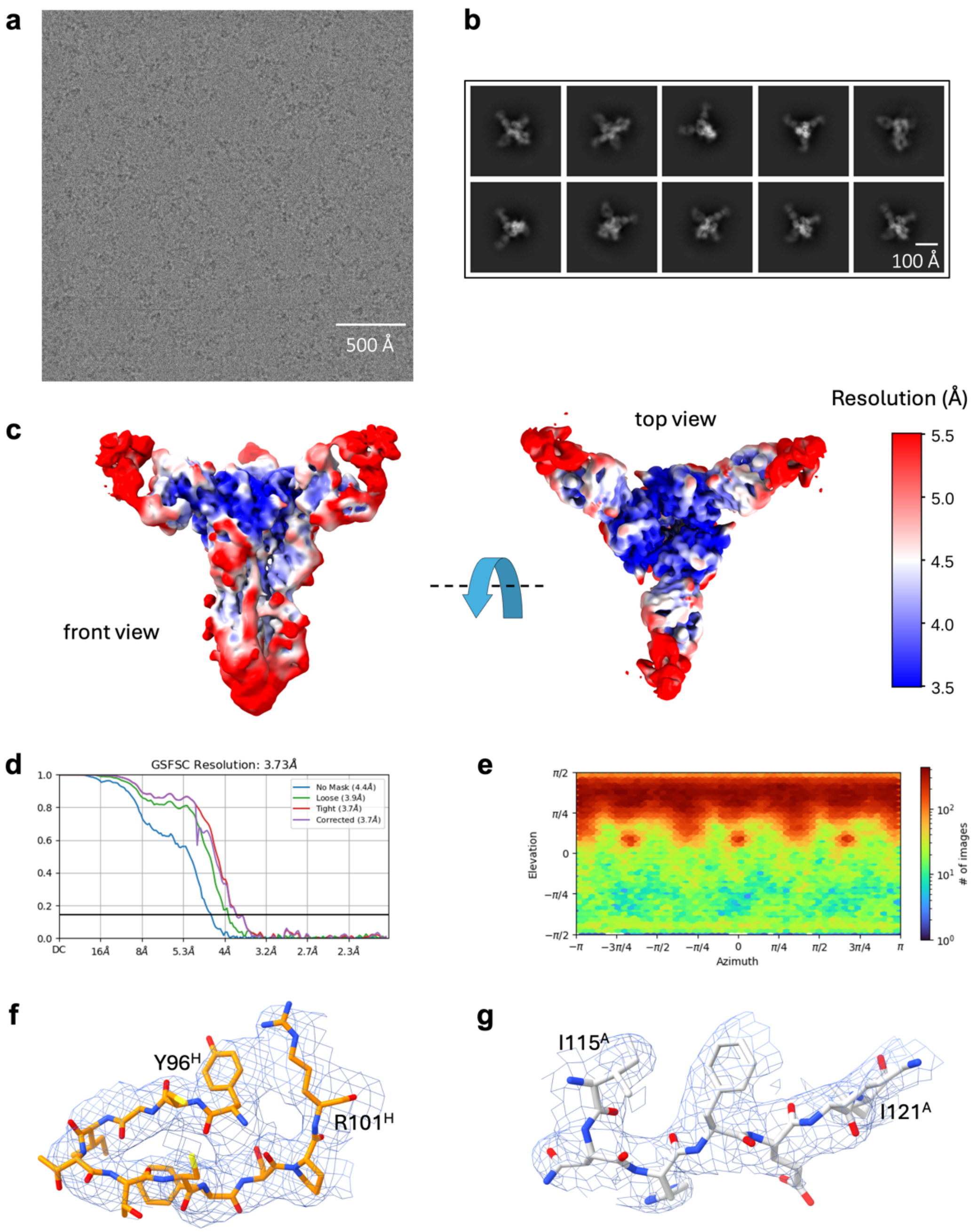

Supplementary Figure 2. Cryo-EM validation of HA in complex with Fab 310-1H02.

a, Representative micrograph. **b**, Representative 2D class averages. **c**, The local resolution estimation of the reconstructed map. **d**, GSFSC resolution curves and **e**, heatmap showing the orientation of the particles used for reconstruction and refinement. **f**, Exemplary cryo-EM map density is shown for select residues of the Fab heavy chain involved in complex formation. **g**, Exemplary cryo-EM density of the residues in HA, chain A. The contour level is 14σ in both panels f and g.

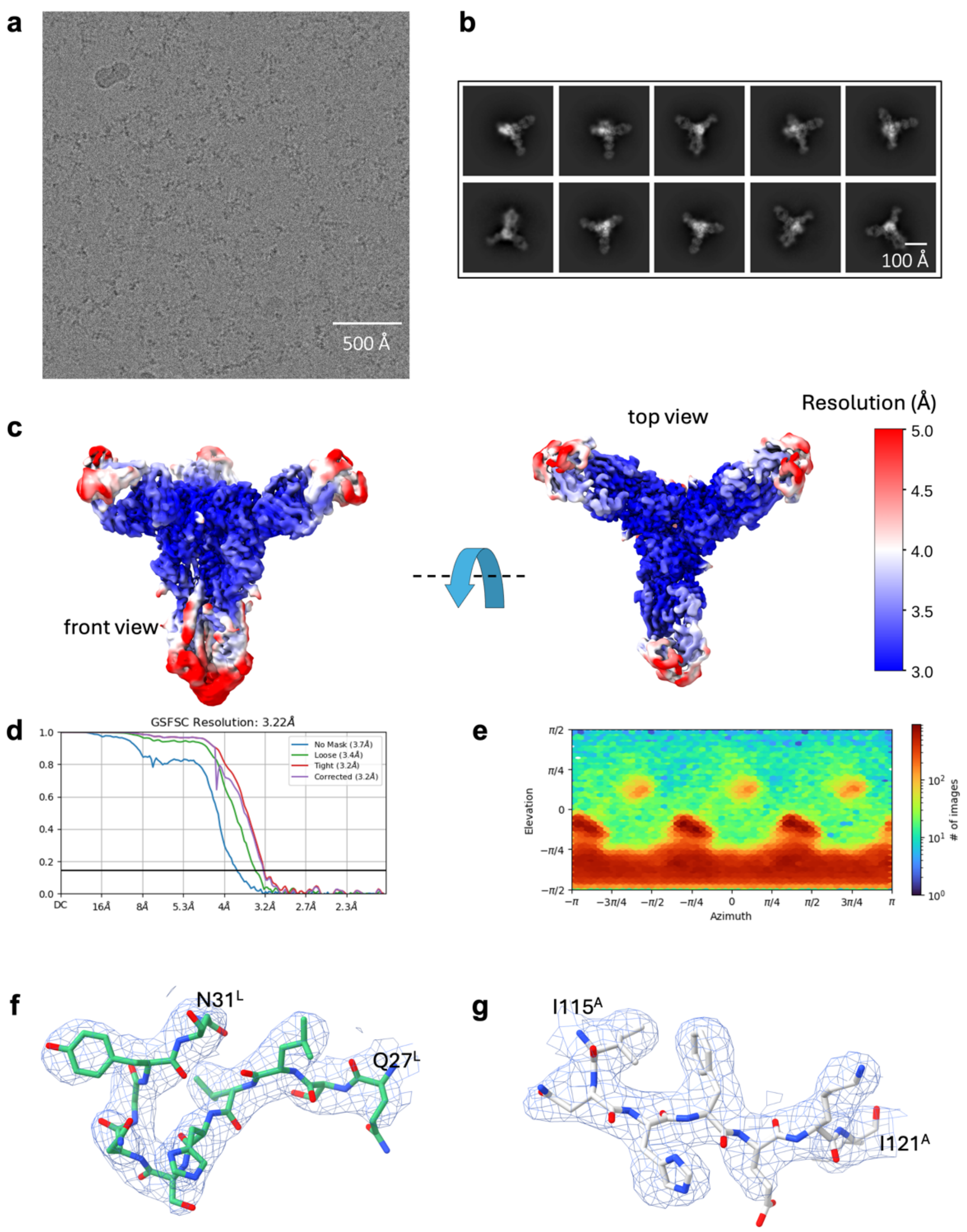

Supplementary Figure 3. Cryo-EM validation of HA in complex with Fab 310-7D11.

a, Representative micrograph. **b**, Representative 2D class averages. **c**, The local resolution estimation of the reconstructed map. **d**, GSFSC resolution curves and **e**, heatmap showing the orientation of the particles used for reconstruction and refinement. **f**, Exemplary cryo-EM map density is shown for select residues of the Fab light chain involved in complex formation. **g**, Exemplary cryo-EM density of the residues in HA, chain A. The contour level is 13σ in both panels f and g.

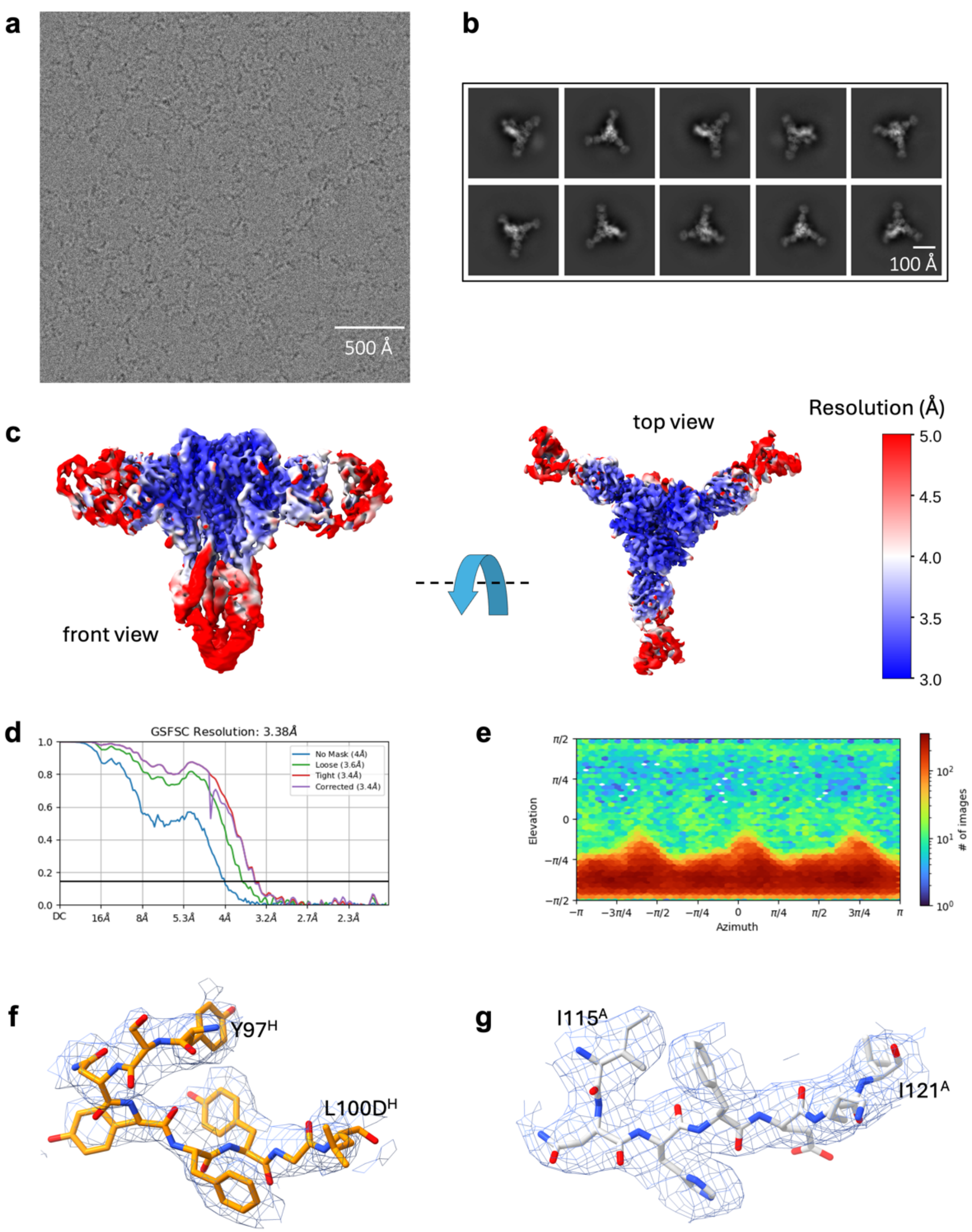

Supplementary Figure 4. Cryo-EM validation of HA in complex with Fab 326-289.74.

a, Representative micrograph. **b**, Representative 2D class averages. **c**, The local resolution estimation of the reconstructed map. **d**, GSFSC resolution curves and **e**, heatmap showing the orientation of the particles used for reconstruction and refinement. **f**, Exemplary cryo-EM map density is shown for select residues of the Fab light chain involved in complex formation. **g**, Exemplary cryo-EM density of the residues in HA, chain A. The contour level is 10σ in both panels f and g.

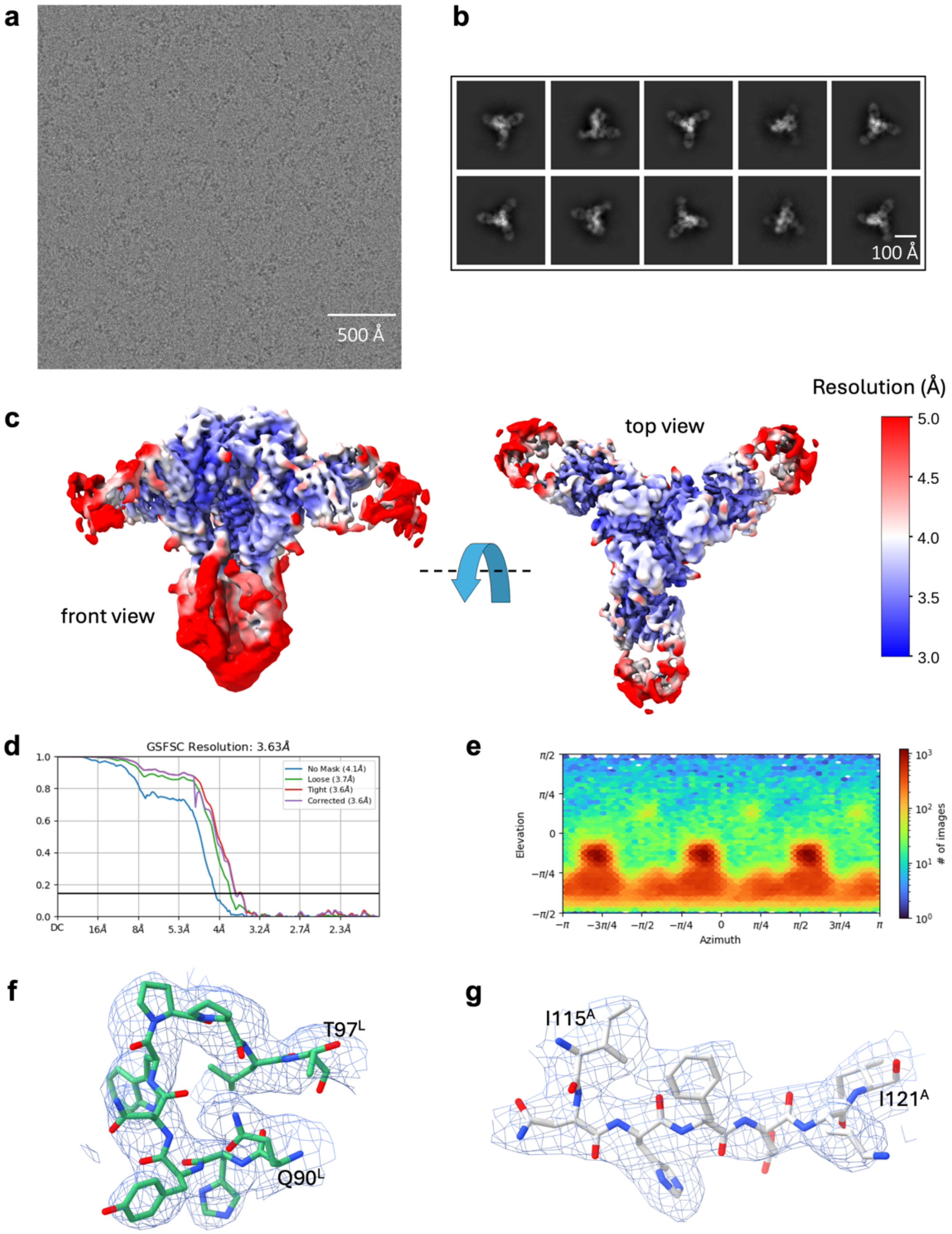

Supplementary Figure 5. Cryo-EM validation of HA in complex with Fab 326-366.26.

a, Representative micrograph. **b**, Representative 2D class averages. **c**, The local resolution estimation of the reconstructed map. **d**, GSFSC resolution curves and **e**, heatmap showing the orientation of the particles used for reconstruction and refinement. **f**, Exemplary cryo-EM map density is shown for select residues of the Fab light chain involved in complex formation. **g**, Exemplary cryo-EM density of the residues in HA, chain A. The contour level is 14σ in both panels f and g.

Supplementary Tables:

Supplementary Table 1: Cryo-EM data collection and single particles analysis statistics

	HA + Fab 326-366.26	HA + Fab 310-1H02	HA + Fab 326-289.74	HA + Fab 310-7D11	HA + Fab 310-12D03
**Data collection and processing**					
Magnification	47,000 x	47,000 x	47,000 x	47,000 x	47,000 x
Microscope	Titan Krios	Titan Krios	Titan Krios	Titan Krios	Titan Krios
Voltage (kV)	300	300	300	300	300
Detector	DE Apollo	DE Apollo	DE Apollo	DE Apollo	DE Apollo
Pixel size (Å)	0.5 (super-resolution)	0.5 (super-resolution)	0.5 (super-resolution)	0.5 (super-resolution)	0.5 (super-resolution)
Electron exposure ($e^{-}/Å^2$)	40.0	40.0	40.0	40.0	40.0
Defocus range (μm)	-1.9 to -0.7	-1.9 to -0.7	-1.9 to -0.7	-1.9 to -0.7	-1.9 to -0.7
Initial particle images (no.)	1,975,843	1,368,467	592,089	1,461,821	1,051,276
Final particle images (no.)	410,566	262,240	181,267	397,663	259,174
Symmetry imposed	C3	C3	C3	C3	C3
Map resolution (Å)	3.76	3.73	3.38	3.22	3.22
FSC threshold	0.143	0.143	0.143	0.143	0.143
**Refinement**					
Model resolution (Å)	4.04	4.16	3.64	3.34	3.38
FSC threshold	0.5	0.5	0.5	0.5	0.5
Map sharpening *B* factor ($Å^2$)	-158.0	-174.6	-114.0	-134.3	-125.0
Model Composition					
Non hydrogen atoms	14,637	14,483	14,723	14,594	14,512
Protein residues	1,861	1,842	1,865	1,895	1,841
B factors ($Å^2$) (mean)	181	228	153	148	146
RMS deviations					
Bond lengths (Å)	0.003	0.003	0.004	0.003	0.003
Bond angles (°)	0.746	0.749	0.798	0.646	0.729
Validation					
MolProbity score	2.00	2.06	1.95	1.55	1.87
Clash score	10.46	12.43	10.53	5.81	8.46
Poor rotamers (%)	0.12	0.37	0.43	0.00	0.31
Ramachandran plot					
Favored (%)	92.6	92.91	93.78	96.42	93.78
Allowed (%)	7.4	6.76	6.11	3.58	6.05
Outliers (%)	0.0	0.34	0.11	0.00	0.17

Supplementary Table 3: SGS frequency by viral HA haplotype

				Selected with 326-289.74					Selected with 310-12D03					
Haplotype	NS mutations	Stock	Cont	R1	R2	R3	R4	R5	R1	R2	R3	R4	R5	R6
Hap5753		98.6	100.0	95.3	59.5	4.9	1.3	0.0	100.0	59.2	30.2	41.1	38.5	0.0
Hap6220	S291N	0.0	0.0	2.9	0.0	0.0	0.0	0.0	0.0	0.0	0.0	0.0	0.0	0.0
Hap4659		0.0	0.0	0.0	0.0	0.0	0.0	0.0	0.0	9.7	28.4	23.9	22.2	0.0
Hap5793	G510E	0.0	0.0	0.0	0.0	0.0	0.0	0.0	0.0	1.4	0.0	0.0	0.0	0.0
Hap6940	G225E	0.0	0.0	0.0	0.0	0.0	0.0	0.0	0.0	0.0	0.0	0.0	7.9	100.0
Hap6790		0.0	0.0	0.0	0.0	0.0	0.0	0.0	0.0	29.7	41.4	35.0	28.7	0.0
Hap9361	D11N	0.0	0.0	0.0	0.0	0.0	0.0	0.0	0.0	0.0	0.0	0.0	2.7	0.0
Hap1670		1.4	0.0	0.0	0.0	0.0	0.0	0.0	0.0	0.0	0.0	0.0	0.0	0.0
Hap5664	K498T	0.0	0.0	0.0	2.7	0.0	0.0	0.0	0.0	0.0	0.0	0.0	0.0	0.0
Hap4900	N169S	0.0	0.0	1.8	0.0	0.0	0.0	0.0	0.0	0.0	0.0	0.0	0.0	0.0
Hap3227	K120T	0.0	0.0	0.0	0.0	0.0	1.2	0.0	0.0	0.0	0.0	0.0	0.0	0.0
Hap3605	K120T	0.0	0.0	0.0	37.7	92.7	97.5	96.6	0.0	0.0	0.0	0.0	0.0	0.0
Hap2095	I60V/K120T	0.0	0.0	0.0	0.0	0.0	0.0	3.4	0.0	0.0	0.0	0.0	0.0	0.0
Hap2349	D90G/K120T	0.0	0.0	0.0	0.0	2.4	0.0	0.0	0.0	0.0	0.0	0.0	0.0	0.0

Supplementary Table 4: SGS frequency by viral HA haplotype after mixed infection

		TX/24 WT + 326-289.74-selected virus					TX/24 WT + 310-12D03-selected virus				
Haplotype	NS mutations	R1	R3.1	R5.1	R3.2	R5.2	R1	R3.1	R5.1	R3.2	R5.2
Hap1670		0.0	0.0	0.0	0.0	0.0	0.0	1.1	0.0	0.0	0.0
Hap12593		0.0	0.0	0.0	2.1	0.0	0.0	0.0	0.0	0.0	0.0
Hap2095	I60V/K120T	4.2	1.9	0.0	0.0	1.6	0.0	0.0	0.0	0.0	0.0
Hap13580	K120T/ R149G	2.0	3.1	3.9	1.8	1.6	0.0	0.0	0.0	0.0	0.0
Hap3605	K120T	65.1	23.7	38.5	22.3	23.2	0.0	0.0	0.0	0.0	0.0
Hap15043	K120T	0.0	0.0	0.0	0.0	4.6	0.0	0.0	0.0	0.0	0.0
Hap16871	T328A	0.0	0.0	0.0	0.0	4.2	0.0	0.0	0.0	0.0	0.0
Hap5696		0.0	0.0	0.0	0.0	7.7	0.0	0.0	0.0	0.0	0.0
Hap17434		0.0	0.0	0.0	0.0	0.0	0.0	0.0	1.0	0.0	0.0
Hap5753		28.7	71.3	57.6	73.9	57.1	42.5	65.1	80.8	65.3	64.3
Hap17600	E468K	0.0	0.0	0.0	0.0	0.0	0.0	0.0	1.5	0.0	0.0
Hap5908	R452K	0.0	0.0	0.0	0.0	0.0	0.0	0.0	2.7	0.0	0.0
Hap17661	L447I	0.0	0.0	0.0	0.0	0.0	0.0	0.0	1.0	0.0	0.0
Hap17735	D419N	0.0	0.0	0.0	0.0	0.0	0.0	0.0	1.0	0.0	0.0
Hap6940	G225E	0.0	0.0	0.0	0.0	0.0	56.1	30.6	12.2	34.7	32.3
Hap19354	G225E/M230I/V377I	0.0	0.0	0.0	0.0	0.0	1.4	1.3	0.0	0.0	3.4
Hap19449	G225E/M230I	0.0	0.0	0.0	0.0	0.0	0.0	1.9	0.0	0.0	0.0

Supplementary Table 5: List of primers used in this study

Step	Oligo type	Oligo name	Sequence (5'-3')
Reverse transcriptase	RT primer	HoffBorRT_12_HA	CCGCTCCGTCCGAACTCACTATANNNNNNNNNNNNAGCRAAAGCAGGGG
PCR	PCR forward	HoffBorPCR-F_Univ	TGGACAGAGTAGAAACAAGG
	Universal PCR reverse	BorPCRRT_Universal	CCGCTCCGTCCGAACTCACTATA
dPCR	dPCR forward	H5-Tex1544Fw	CCKCAGTATTCAGAAGAAGC
	dPCR reverse	H5-1683Rv	AGACCAGCYAYCATGATTGC
	dPCR Probe	H5d-1638TexProbe(+)	/56-FAM/AGTGCTAGRGAACTYGCMRCTGTWG/3BHQ_1/
