# Supplementary material for: Cross-neutralizing and potent human monoclonal antibodies against historical and emerging H5Nx influenza viruses
Source: Nat Microbiol. 2025 Oct 14;10(11):2903–18. doi: 10.1038/s41564-025-02137-x (PMC12578633; doi:10.1038/s41564-025-02137-x)
Supplement: Supplementary file 2 — Reporting Summary [file 41564_2025_2137_MOESM2_ESM.pdf]

nature portfolio

Corresponding author(s): Tongqing Zhou, Masaru Kanekiyo, and Sarah Andrews

Last updated by author(s): Aug 15, 2025

Reporting Summary

Nature Portfolio wishes to improve the reproducibility of the work that we publish. This form provides structure for consistency and transparency in reporting. For further information on Nature Portfolio policies, see our Editorial Policies and the Editorial Policy Checklist.

Statistics

For all statistical analyses, confirm that the following items are present in the figure legend, table legend, main text, or Methods section.

n/a	Confirmed	
☐	☒	The exact sample size (*n*) for each experimental group/condition, given as a discrete number and unit of measurement
☐	☒	A statement on whether measurements were taken from distinct samples or whether the same sample was measured repeatedly
☐	☒	The statistical test(s) used AND whether they are one- or two-sided<br>*Only common tests should be described solely by name; describe more complex techniques in the Methods section.*
☒	☐	A description of all covariates tested
☐	☒	A description of any assumptions or corrections, such as tests of normality and adjustment for multiple comparisons
☐	☒	A full description of the statistical parameters including central tendency (e.g. means) or other basic estimates (e.g. regression coefficient) AND variation (e.g. standard deviation) or associated estimates of uncertainty (e.g. confidence intervals)
☐	☒	For null hypothesis testing, the test statistic (e.g. *F*, *t*, *r*) with confidence intervals, effect sizes, degrees of freedom and *P* value noted<br>*Give P values as exact values whenever suitable.*
☒	☐	For Bayesian analysis, information on the choice of priors and Markov chain Monte Carlo settings
☒	☐	For hierarchical and complex designs, identification of the appropriate level for tests and full reporting of outcomes
☒	☐	Estimates of effect sizes (e.g. Cohen's *d*, Pearson's *r*), indicating how they were calculated

Our web collection on statistics for biologists contains articles on many of the points above.

Software and code

Policy information about availability of computer code

Data collection	FACSDiva v8<br>SerialEM 4.09
Data analysis	FlowJo v10<br>bclfastq v2.20.0.422<br>SONAR v4.3<br>MEGA11<br>Skylign<br>GraphPad Prism v10<br>polyclonal package (https://jbloomlab.github.io/polyclonal/)<br>Perl v5.16.3<br>RStudio v2022.07.1<br>Minimap2<br>SeqKit v2.3.1<br>Cutadapt v4.0<br>MAFFT v7.467<br>Geneious Prime v2023.0.4<br>Carterra Kinetics<br>Topaz<br>Relion<br>cryoSPARC

deepEMhancer
ColabFold
Coot
Phenix
ISOLDE
UCSF ChimeraX

For manuscripts utilizing custom algorithms or software that are central to the research but not yet described in published literature, software must be made available to editors and reviewers. We strongly encourage code deposition in a community repository (e.g. GitHub). See the Nature Portfolio guidelines for submitting code & software for further information.

Data

Policy information about availability of data

All manuscripts must include a data availability statement. This statement should provide the following information, where applicable:

- Accession codes, unique identifiers, or web links for publicly available datasets
- A description of any restrictions on data availability
- For clinical datasets or third party data, please ensure that the statement adheres to our policy

The cryo-EM maps and atomic coordinates of the H5N1 TX/24 HA –Fab complexes for mAbs 326-366.26, 310-1H02, 326-289.74, 310-7D11, and 310-12D03 have been deposited in the Electron Microscopy Data Bank (EMDB) and the Protein Data Bank (PDB). The corresponding EMDB accession codes are EMD-48515, EMD-48516, EMD-48517, EMD-48518, and EMD-48521, while the PDB accession codes for the atomic coordinates are 9MQ7, 9MQ8, 9MQ9, 9MQA, and 9MQD, respectively. High-throughput SGS data are deposited to NCBI BioProject under accession PRJNA1220181. All data, analysis and figures related to deep mutational scanning experiments have been archive on Zenodo under DOI:10.5281/zenodo.1674086276. Deep mutational scanning analysis pipeline and data is also publicly available on Github at https://github.com/dms-vep/Flu_H5_American-Wigeon_South-Carolina_2021-H5N1_DMS. Nucleotide sequences for mAbs are in Genbank accession numbers PX104069-PX104338. Requests for materials should be addressed to the corresponding authors. mAbs under patent can be provided with a material transfer agreement.

Research involving human participants, their data, or biological material

Policy information about studies with human participants or human data. See also policy information about sex, gender (identity/presentation), and sexual orientation and race, ethnicity and racism.

Reporting on sex and gender	Sex information is available in Extended Data Table 1.
Reporting on race, ethnicity, or other socially relevant groupings	Race and ethnicity was collected as part of the original Phase I clinical trial from which samples were obtained, but is not reported here as is irrelevant to this post-hoc study
Population characteristics	For the H5 vaccine trial healthy adults aged 18 to 60 years were enrolled. In the FluMos-v2 vaccine trial healthy adults born between 18 and 50 were enrolled. Inclusion criteria for both trials required general good health determined by laboratory tests, medical history, and physical exam.
Recruitment	Volunteers were recruited from the greater Washington, DC, area by IRB-approved written and electronic media.
Ethics oversight	The trial protocol was reviewed and approved by the NIAID Institutional Review Board. U.S. Department of Health and Human Services guidelines for conducting clinical research were followed.

Note that full information on the approval of the study protocol must also be provided in the manuscript.

Field-specific reporting

Please select the one below that is the best fit for your research. If you are not sure, read the appropriate sections before making your selection.

☒ Life sciences ☐ Behavioural & social sciences ☐ Ecological, evolutionary & environmental sciences

For a reference copy of the document with all sections, see nature.com/documents/nr-reporting-summary-flat.pdf

Life sciences study design

All studies must disclose on these points even when the disclosure is negative.

Sample size	Sample sizes of animal studies were determined based on prior experience with similar experiments. Assuming variance in the lethality is proportional to mean for a given group (constant CV of 30%, typical for this type of experiments), a group size of 10 will give 89% power to detect 2-fold differences or a 49% power to detect 1.5-fold differences between groups based on a two tailed test of means with an alpha set to 0.05 (calculation was performed by 1-way ANOVA pairwise tools at powerandsamplesize.com).
Data exclusions	No data were excluded.
Replication	Structural determination of HA-Fab complexes was performed once as is custom for CryoEM. All in vitro assays were repeated at least twice

Replication	with similar results. Animal infection studies were performed once as they are BSL-3 and resource intensive.
Randomization	All animals used in the study were randomly assigned to different experimental groups.
Blinding	All in vitro experiments were not performed blindly as samples were labeled with mAb names. However, data collected is quantitative measured by instrumentation, so not open to interpretation or require judgement calls. All in vivo experiments were performed blindly to animal handlers.

Reporting for specific materials, systems and methods

We require information from authors about some types of materials, experimental systems and methods used in many studies. Here, indicate whether each material, system or method listed is relevant to your study. If you are not sure if a list item applies to your research, read the appropriate section before selecting a response.

Materials & experimental systems

n/a	Involved in the study	
☐	☒	Antibodies
☐	☒	Eukaryotic cell lines
☒	☐	Palaeontology and archaeology
☐	☒	Animals and other organisms
☐	☒	Clinical data
☒	☐	Dual use research of concern
☒	☐	Plants

Methods

n/a	Involved in the study	
☒	☐	ChIP-seq
☐	☒	Flow cytometry
☒	☐	MRI-based neuroimaging

Antibodies

Antibodies used	CD19 BV750, BD#747161, clone SJ25-C1, 1:400 dilution<br>IgG BUV395, BD#564229, clone G18-145, 1:200 dilution<br>IgM BB700, custom, BD, clone G20-127, 1:400 dilution<br>CD3 BV510, Biolegend#317332, clone OKT3, 1:400 dilution<br>CD14 BV510, Biolgend#301842, clone M5E2, 1:200 dilution<br>CD56 BV510, Biolegend# 318340, clone HCD56, 1:200 dilution<br>CD20 APC-Cy7, Biolegend#302313, clone 2H7, 1:400 dilution<br>CD27 BV605, Biolegend#302830, clone O323, 1:100 dilution<br>CD21 PE594, BD#563474, clone B-ly4, 1:400 dilution<br>IgA APC, Miltenyi #130-113-472, clone IS11-8E10, 1:400 dilution
Validation	The technical data sheets from the manufacturer for all antibodies state that they are specifically tested and validated to bind the human antigen listed either through testing on human PBMCs or cell lines expressing the antigen of interest. All antibodies were titered to determine optimal amounts to maximize signal to noise.

Eukaryotic cell lines

Policy information about cell lines and Sex and Gender in Research

Cell line source(s)	Expi293 (ThermoFisher A14527),MDCK-SIAT1-PB1 (Creanga et al. Nat Commun. 2021), 293T (ATCC), MDCK (ATCC, CCL-34)
Authentication	Cell lines were not authenticated.
Mycoplasma contamination	Tested negative (monthly).
Commonly misidentified lines<br>(See ICLAC register)	n/a

Animals and other research organisms

Policy information about studies involving animals; ARRIVE guidelines recommended for reporting animal research, and Sex and Gender in Research

Laboratory animals	BALB/c mice
Wild animals	n/a

Reporting on sex	Passive transfer study used only female mice because of the previous LD50 titration studies were performed by using female mice. Pharmacokinetics used a mixture of male and female mice.
Field-collected samples	n/a
Ethics oversight	All experiments were conducted in accordance with the National Institutes of Health (NIH) recommendations in the Guide for the Care and Use of Laboratory Animals with pre-approval of specific procedures and protocols by the Institutional Animal Care and Use Committee of the University of Pittsburgh, with animal care in accordance with the Association for Assessment and Accreditation of Laboratory Animal Care (AAALAC). All mice in this study were housed in AAALAC accredited animal facilities in a 12-hour light/dark cycle at an ambient temperature of 22.2 ± 2.8ºC with a relative humidity maintained between 30–70%.

Note that full information on the approval of the study protocol must also be provided in the manuscript.

Clinical data

Policy information about clinical studies
All manuscripts should comply with the ICMJE guidelines for publication of clinical research and a completed CONSORT checklist must be included with all submissions.

Clinical trial registration	NCT01086657 and NCT05968989
Study protocol	Study details can be found under clinical trial registrations noted above.
Data collection	N/A - this is post-hoc use of study samples, not report of clinical study results
Outcomes	N/A - this is post-hoc use of study samples, not report of clinical study results

Plants

Seed stocks	*Report on the source of all seed stocks or other plant material used. If applicable, state the seed stock centre and catalogue number. If plant specimens were collected from the field, describe the collection location, date and sampling procedures.*
Novel plant genotypes	*Describe the methods by which all novel plant genotypes were produced. This includes those generated by transgenic approaches, gene editing, chemical/radiation-based mutagenesis and hybridization. For transgenic lines, describe the transformation method, the number of independent lines analyzed and the generation upon which experiments were performed. For gene-edited lines, describe the editor used, the endogenous sequence targeted for editing, the targeting guide RNA sequence (if applicable) and how the editor was applied.*
Authentication	*Describe any authentication procedures for each seed stock used or novel genotype generated. Describe any experiments used to assess the effect of a mutation and, where applicable, how potential secondary effects (e.g. second site T-DNA insertions, mosiacism, off-target gene editing) were examined.*

Flow Cytometry

Plots

Confirm that:

☒ The axis labels state the marker and fluorochrome used (e.g. CD4-FITC).

☒ The axis scales are clearly visible. Include numbers along axes only for bottom left plot of group (a 'group' is an analysis of identical markers).

☒ All plots are contour plots with outliers or pseudocolor plots.

☒ A numerical value for number of cells or percentage (with statistics) is provided.

Methodology

Sample preparation	All samples were viably frozen PBMCs thawed at 37C and stained immediately with cell surface markers for analysis and sorting. Cell viability was always above 95%.
Instrument	All data was collected on a BD FACSymphony S6
Software	Data collection was performed with BD FACSDiva v.8, data analysis was done with FlowJo v.10
Cell population abundance	All sorting was single-cell into individual wells in 96-well plates. Confirmation that B cells were sorted was the ability to amplify immunoglobulin sequences from each well. A subset of immunoglobulin sequences were used to produce monoclonal antibodies that validated specificity.

Gating strategy

Provided as Extended Data Figure 1

☒ Tick this box to confirm that a figure exemplifying the gating strategy is provided in the Supplementary Information.
